# Blood Plasma’s Protective Ability against the Degradation of S-Nitrosoglutathione under the Influence of Air-Pollution-Derived Metal Ions in Patients with Exacerbation of Heart Failure and Coronary Artery Disease

**DOI:** 10.3390/ijms221910500

**Published:** 2021-09-28

**Authors:** Anna Wądołek, Dominika Drwiła, Maria Oszajca, Grażyna Stochel, Ewa Konduracka, Małgorzata Brindell

**Affiliations:** 1 Faculty of Chemistry, Jagiellonian University in Kraków, Gronostajowa 2, 30-387 Krakow, Poland; anna.wadolek@doctoral.uj.edu.pl (A.W.); maria.oszajca@uj.edu.pl (M.O.); grazyna.stochel@uj.edu.pl (G.S.); 2 Department of Coronary Disease and Heart Failure, John Paul II Hospital, 31-202 Krakow, Poland; dominika.drwila@gmail.com; 3 Department of Coronary Disease and Heart Failure, Jagiellonian University Medical College, John Paul II Hospital, 31-202 Krakow, Poland

**Keywords:** air pollution, particulate matters (PM), metal ions, S-nitrosothiols, albumin, S-nitrosoglutathione, nitric oxide, ascorbic acid, cardiovascular diseases

## Abstract

One of the consequences of long-term exposure to air pollutants is increased mortality and deterioration of life parameters, especially among people diagnosed with cardiovascular diseases (CVD) or impaired respiratory system. Aqueous soluble inorganic components of airborne particulate matter containing redox-active transition metal ions affect the stability of S-nitrosothiols and disrupt the balance in the homeostasis of nitric oxide. Blood plasma’s protective ability against the decomposition of S-nitrosoglutathione (GSNO) under the influence of aqueous PM extract among patients with exacerbation of heart failure and coronary artery disease was studied and compared with a group of healthy volunteers. In the environment of CVD patients’ plasma, NO release from GSNO was facilitated compared to the plasma of healthy controls, and the addition of ascorbic acid boosted this process. Model studies with albumin revealed that the amount of free thiol groups is one of the crucial factors in GSNO decomposition. The correlation between the concentration of NO released and -SH level in blood plasma supports this conclusion. Complementary studies on gamma-glutamyltranspeptidase activity and ICP-MS multielement analysis of CVD patients’ plasma samples in comparison to a healthy control group provide broader insights into the mechanism of cardiovascular risk development induced by air pollution.

## 1. Introduction

Many clinical and epidemiological studies emphasize the negative impact of air pollution on morbidity, mortality, and deterioration of human health. As indicated by the WHO, as many as 23% of deaths worldwide occur due to modifiable environmental factors [1]. Based on the exposure assessment and evidence-based medicine research, the top of the list of the most dangerous environment-related diseases are stroke, ischemic heart disease, diarrhea, lower respiratory tract infections, allergies, and cancer, most of which are closely related to the inhalation of polluted air. Cardiovascular diseases (CVD) are a group of disorders particularly vulnerable to harmful effects of particulate matter (PM) in terms of mortality and deterioration of life parameters [2]. Breathing air with high concentrations of suspended PM_2.5_ and PM_10_ dust particles has been shown to correlate with an increased incidence of high-risk cardiovascular events such as atherosclerosis, ischemic stroke, hypertension, or thromboembolic complications. In addition, it has been proven that air pollution not only initiates the emergence of cardiovascular diseases but also intensifies the course of existing diseases [3]. Numerous case-control studies have confirmed the relationship between individual disease entities and exposure to air pollutants, although the detailed mechanism of their impact has not been elucidated.

Disturbance in nitric oxide (NO) bioavailability is thought to be one of the key factors common to cardiovascular disease [4]. Among others, appropriate delivery of NO is crucial for vasodilation effect via cGMP-dependent activation of smooth muscle cells which prevents hypertension [5]. Furthermore, endothelium-derived NO prevents platelet adhesion and endothelial cell apoptosis, induced by proinflammatory cytokines and proatherosclerotic factors, including reactive oxygen species and angiotensin II [6]. The suppression of apoptosis may also contribute to the anti-inflammatory and antiatherosclerotic effects of endothelium-derived NO. On the other hand, the uncontrolled elevation of the NO concentration may exacerbate inflammation through the damage of the endothelial cells, mediated by superoxide anion and hydroxyl radical, local vasoconstriction, and increased vascular resistance as well as aggregation and growth of platelets [7,8]. S-nitrosothiols serve as a circulating source of stored NO with the capacity to be released enzymatically or nonenzymatically through various factors such as metal ions or reactive oxygen species [9]. Therefore, it is of great importance to control the bioavailability of S-nitrosothiols, which play a significant role in mediating NO bioactivity [10,11,12,13]. Human plasma, which is rich in proteins, enzymes, and antioxidants, can prevent the uncontrolled decomposition of S-nitrosothiols caused by external degradative factors, among others [14].

There are many changes in the composition of blood plasma of patients with CVD compared to the healthy ones. One of the effects of CVD is the depletion of plasma albumin accompanied by its qualitative changes, such as oxidation of -SH groups, which results in the accessibility of free thiols in plasma [15,16,17,18,19,20,21,22,23,24]. Furthermore, plasma γ-glutamyl transpeptidase (GGTP) involved in glutathione metabolism has been marked in several population-based studies to be associated with the occurrence of CVD and increase in the risk of death [25,26,27,28]. Hyperactivity of this enzyme contributes to the production of reactive oxygen species through modulating the redox status of cell surface protein thiols, causing lipid peroxidation and endothelial cell damage [29,30]. Furthermore, clinical study revealed enhanced activity of xanthine oxidase (XO) associated with progressive circulatory system disease [31]. XO has a strong affinity toward low molecular weight nitrosothiols such as S-nitrosoglutathione (GSNO) or S-nitrosocystein (CysNO), catalyzing their degradation via superoxide dependent pathway [32]. Further, the impairment of the activity of S-nitrosoglutathione reductase and thioredoxin reductase systems, important regulators of denitrosylation, has been outlined as a pathological factor in cardiovascular diseases [33,34]. All these changes may adversely affect the protective ability of blood plasma in CVD patients.

Blood plasma can also be modified due to organism exposure to air pollutants. One of the components of suspended PM_2.5_ and PM_10_ particles are inorganic compounds containing transition metal ions that can penetrate into the bloodstream and disturb the physiological redox balance [35,36]. Transition metal ions in the plasma are scavenged by high-weight proteins mainly human serum albumin (HSA) but also ceruloplasmin or transferrin to prevent their uncontrolled auto-oxidation and generation of reactive oxygen species [37]. The metal ion binding capacity of HSA can be altered by binding fatty acid chains, which leads to an allosteric switch in the protein structure [38]. It was shown that among patients diagnosed with CVD, a common clinical symptom is an increase in the levels of triglyceride and low-density lipoproteins and as a consequence, an increased amount of fatty acids is coupled with albumin [24]. Several important biological functions of HSA (nitric oxide transport, antioxidant properties, and metal-binding capacity) are related to the accessibility of the free thiol group in Cys34. Since Cys34 can readily respond to transition metal ions (either by binding or oxidation), [39] its function can be disturbed by the exposure of the plasma to pollutants. Furthermore, the negative influence of the PM-derived transition metal ions on the stability of S-nitrosoalbumin (HSA-NO) and low-molecular-weight S-nitrosothiols was also demonstrated [40,41]. Moreover, metal ions originating from the inhaled PM, after encountering the lung lining fluid containing a substantial concentration of ascorbic acid, may change their oxidation state and potency to decompose S-nitrosothiols. Even though ascorbic acid is a common and important free radical scavenger, positively associated with reduced risk of cardiovascular events, it should be kept in mind that it can have both a pro- and antioxidant nature [42]. It can be readily oxidized by undergoing one-electron transfer in the presence of transition metal ions especially Cu^2+^, generating reactive oxygen species hydroxyl radical (OH^•^) and hydrogen peroxide (H_2_O_2_) [43]. These, in turn, are known for causing oxidative damage of lipoproteins. After such modification, their uptake in the vascular walls is facilitated, causing endothelial injury and further disruption of the nitric oxide bioavailability, leading to further CVD progression [44].

The changes in blood plasma, induced by soluble constituents of airborne particulate matter, can among others, affect S-nitrosothiol stability and deregulate NO-based biological signaling both in CVD and healthy subjects. However, due to some pathological changes in the plasma of the CVD patients, the response to the exposure to the pollutant might be different than healthy subjects. Difficulties in researching the molecular bases showing a link between the progression of CVD and the exposure to PMs arise from the variety of factors leading to the development of the diseases and the fact that the observed effects are associated with chronic exposure to harmful substances, which is difficult to imitate in laboratory conditions. Therefore, to obtain any information about the impact of air pollution on CVD, there is a need to design experimental models enabling the identification of the molecular processes that might be responsible for the observed outcomes. So, in this report we focus on confirming some of the qualitative changes in the plasma caused by the occurrence of chronic heart failure exacerbation (CHFE) or significant coronary artery disease (CAD) and examination whether these changes affect the protective ability of blood plasma against the degradation of S-nitrosoglutathione under the influence of pollution derived metal ions. The results for CHFE and CAD patients are compared with results obtained for the healthy control group and supported with the model studies developed by us. We believe that the results reported herein might contribute to a better understanding of the molecular aspects of air pollution-related CVD progression.

## 2. Results and Discussion

### 2.1. Influence of Urban PM Extracts on the GSNO Decomposition in the Blood Plasma Environment

The analysis of the blood plasma protective ability against the destabilization of S-nitrosothiols by the water-soluble inorganic fraction of particulate matter was performed in the model systems described below. Blood plasma obtained from three groups of patients diagnosed with CHFE or CAD and convergent healthy controls was used for the experiments. For each plasma sample, the fixed concentration of GSNO (5 µM) was injected into 5 mL of a solution containing 10% vol. of urban PM extracts in the blood plasma solution obtained by twenty-fold dilution with Tris buffer. The amount of the PM extract to be applied was determined experimentally in such a way that it was fixed at the lowest possible level, but still allowing the detection of differences in relation to the samples not treated with PM.

**Figure 1:**
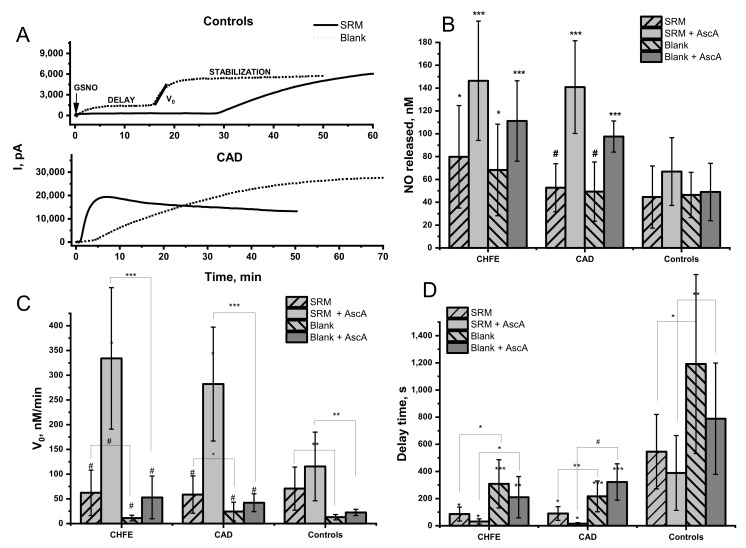
NO release from GSNO in 20-fold diluted blood plasma environment from CHFE (*n* = 7), CAD (*n* = 8) and controls (*n* = 15) participants in the absence (blank) or the presence of 10% vol. of SRM aqueous extract without or with the addition of 1 µM of ascorbic acid (AscA). Measurements were performed using NO electrode in Tris buffer 0.1 M, pH = 7.4, T = 37 °C in the presence of 5µM of GSNO, in the dark. Measurements of the samples were performed in triplicates. The data represent the mean ± SD. The values of * *p* < 0.05, ** *p* < 0.01, *** *p* < 0.001 vs. controls, were considered significant; # *p* > 0.05. (**A**) Typical NO release profiles (**A**) and the calculated: the mean amount (**B**), the initial rates (**C**) and the delay time (**D**) of NO release.

The amount of released NO was registered with the selective NO sensor and the registered current difference was converted into actual NO concentration using a standard calibration curve. A typical evolution of the signal is shown in Figure 1A. The impact on the NO release from GSNO was studied under physiological-like conditions, that is pH 7.4 and 37 °C. The obtained data were analyzed for the total amount of NO released from GSNO determined after reaching the signal saturation on the curve (Figure 1B). Additionally, the initial reaction rate (Figure 1C) and the delays in the current signal growth after GNSO injection (Figure 1D) were analyzed.

In the environment of blood plasma, GSNO decomposition level was higher in the CVD patients’ groups than in the healthy controls. The amount of NO released in the CHFE patients’ group was significantly higher when compared to the control group. The addition of SRM extract to the blood plasma solution led to the release from 1.5% to 0.8% of NO from the total pool of available GSNO, depending on the study group (Figure 1B). The relatively high signal registered for the decomposition of GSNO in a neat blood plasma environment (without SRM extract) cannot be omitted. The amount of NO released from GSNO in a neat plasma compared to SRM extract-treated one was nonsignificantly lower within each studied group but was still remarkably higher in CHFE vs. healthy controls. (*p* = 0.03).

It should be noted that the initial rate of decomposition of GSNO in the neat plasma environment was meaningfully lower than for NO release caused by metal ions present in the SRM extract (Figure 1C), therefore these two cases should not be considered competitive. Furthermore, the initial rate of GSNO decomposition in neat plasma was higher in samples from CHFE participants than for CAD and healthy control, which pointed to the differences in the plasma composition. An intriguing parameter requiring analysis was the time delay between the GSNO injection and the actual signal growth registered for NO signal development. In our previous model studies, an immediate increase in the current signal was registered after S-nitrosothiols injection to the studied reaction solution [40,41]. A significant difference in the delay time was observed for the plasma samples from CVD subjects vs. healthy control (*p* = 0.004 and *p* < 0.001 for CHFE and CAD, respectively). It may indicate that plasma from healthy participants plays a protective role against an uncontrolled, fast, and excessive increase in NO level. The time needed to start NO signal growth was ca. 20 min in neat plasma from healthy control and 4–5 min in plasma of CVD participants. The addition of the SRM extract reduced the time delay by more than half. Thus, despite the slight differences in the amount of NO released from GSNO detected in the plasma environment of CVD participants and healthy control, a strong protective role of plasma from healthy control is manifested in pronounced delay and the reduced initial rate of NO release. This effect is even more evident in the presence of pollutants.

Several factors may be responsible for the observed differences in the profile of NO release in blood plasma environment from healthy control compared to CVD patients’ groups as well as for the impact of the SRM extract on this process. In our previous study, it was shown that reducing agents such as ascorbic acid (AscA) and GSH, as well as metal ions (copper in particular), can accelerate the decomposition of GSNO in aqueous solutions and may modulate the release of NO from S-nitrosothiols [42]. In further chapters of this publication, we show how supplementation with vitamin C (ascorbic acid, AscA) and changes in (i) free thiol availability, (ii) γ-glutamyl transpeptidase activity, and (iii) metal ions content; influence the alteration in GSNO decomposition in the studied blood plasma environments.

### 2.2. Effect of Ascorbic Acid on the GSNO Decomposition by Urban PM Extracts in the Blood Plasma Environment

Currently, it is known that oxidative stress plays a crucial role in CVD pathophysiology [45], and to decrease its level, supplementation with vitamin C (ascorbic acid, AscA), a well-known antioxidant, is proposed among others [46,47,48]. Unfortunately, the determination of vitamin C in blood samples is quite challenging due to its fast oxidation and further metabolization as well as degradation ex vivo [49]. To get any information about the potential role of AscA supplementation in GSNO decomposition in blood plasma environment its external addition (1 µM) was employed. The addition of AscA into the blood plasma solution containing the SRM extract immediately before GSNO injection resulted in a high increase of the signal from the NO sensor (Figure 1B). In both CHFE and CAD groups, decomposition of GSNO was boosted compared to healthy controls (*p* = 0.0006; *p* = 0.0002) and plasma not supplemented with AscA (Figure 1B). Moreover, within the groups of CVD patients (but not healthy controls), the amount of NO released by the SRM extract pretreated with AscA was significantly more pronounced than in the case of AscA on its own.

The GSNO decomposition in the presence of AscA was accelerated compared to samples not supplemented with AscA in all examined groups (Figure 1C). The addition of AscA into the blood plasma solution containing SRM extract resulted in a significant increase in the initial rate of NO release in plasma from CVD vs. control (*p* = 0.0012 and *p* < 0.001 for CHFE and CAD, respectively). Within each studied group in which the plasma was supplemented with AscA, the initial rate of NO release from GSNO was significantly higher in the presence of SRM extract (*p* < 0.001, *p* < 0.001, and *p* = 0.002, for CHFE, CAD, and control respectively). Furthermore, the delay time in NO release was significantly reduced by the addition of AscA, either to neat plasma or plasma containing SRM extract in the CVD group vs. control. It may point to a decreased protective ability of plasma altered by CVD against exposure to pollutants.

It was previously shown that the reduced form of the copper ion (Cu^+^) is mainly responsible for S-nitrosothiols decomposition in the presence of SRM extract pretreated with AscA [40,41,50]. The observed strongly elevated and accelerated release of NO from GSNO in plasma environment from CVD participants compared to the control may be due to the ineffective binding the SRM extract derived transition metal ions, in particular redox-active copper ions. The lower metal scavenging capacity in patients diagnosed with CVD may be due to the reduced amount of plasma albumin [21,23,51,52,53] and the decreased content of available free thiols (discussed in the next section). The qualitative changes of HSA caused (among others) by the binding of fatty acids can also be responsible [20].

The pro-oxidant nature of AscA may occur in the presence of transition metal ions leading to the formation of ascorbyl, nitroxyl, or OH^•^ radicals, which cannot be efficiently scavenged [35,54]. This can result in a further increase in the NO release yield, its acceleration, and a decrease in the delay time due to the radical nature of the process. Therefore, a much higher increase of the NO signal observed for the plasma environment of CVD patients in the presence of SRM extract and AscA, compared to the healthy control, may also arise from impaired prevention against formation and neutralization of reactive oxygen/nitrogen species. The level of extracellular superoxide dismutase (EC-SOD) was noticeably decreased in patients diagnosed with CVD [55], which can result in a weaker neutralization of superoxide radical and decomposition of nitroxyl. Both influence the NO release from GSNO [42,54].

### 2.3. Free Thiol Content in Human Plasma and Its Influence on NO Release from GSNO

**Figure 2:**
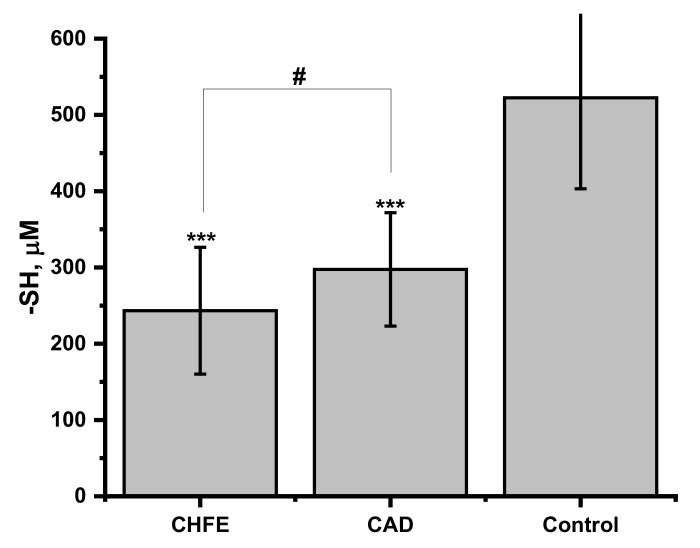
The assessment of free thiol groups in blood plasma from CHFE (*n* = 7), CAD (*n* = 8) and control (*n* = 15) participants. Measurements of the samples were performed in triplicates. The data represent mean ± SD. The values of *** *p* < 0.001 vs. controls, were considered significant; # *p* > 0.05.

As the pool of free thiol groups might be a differentiating factor in the protective ability of blood plasma against S-nitrosoglutathione (GSNO) degradation by pollution-derived metal ions, the total thiols concentration in the studied blood plasma samples was determined by Ellman’s method (Figure 2). The significantly lower free-thiols level was determined in the plasma of CHFE and CAD groups when compared to the healthy controls (*p* = 0.00003 and 0.0001, respectively). However, for both groups of CVD participants similar levels of -SH (*p* = 0.24 for CHFE vs. CAD) was determined. These results are in line with the current literature reports showing that in many case-control and cohort studies, decreased levels of native thiol were associated with the presence and severity of heart failure or coronary artery disease [21,51,52,53]. Abdulle et al. indicated it as an important predictive factor in the risk of hazardous cardiovascular events and in all-cause mortality in the general population [23]. The decrease in the number of -SH groups may arise from their involvement in the neutralization of the reactive oxygen species resulting from the increased oxidative stress observed in patients with CVD [45].

One of the pathways of thiol-related circulatory system pathology progression might be caused by the enhanced release of NO from S-nitrosothiols. Therefore, the examination of the strength and association between measured free thiol concentration and the amount of NO released in human plasma samples from all participants was calculated using Pearson’s correlation test (Table 1). A strong, negative relationship between GSNO decomposition and -SH concentration occurred under the conditions of plasma environment with the addition of ascorbic acid both in the presence and absence of SRM (r = −0.75, *p* < 0.001; r = −0.71, *p* < 0.001, respectively). NO released from GSNO under the influence of SRM extracts ions added to plasma showed a weak, but still significant, negative correlation with thiols level (r = −0.39; *p* = 0.035). No correlation between self-decomposition of GSNO and -SH level was detected for neat blood plasma samples. These results pointed out the significant role of free thiol groups in the GSNO decomposition through scavenging metal ions originating from SRM extract [35,37] as well as a protective role against the effects of elevated levels of ascorbic acid. Limited availability of free thiols may also result in reduced generation of more stable species in the transnitrosylation reaction (see Section 2.5).

### 2.4. Effect of Free Thiol Groups in Albumin on GSNO Decomposition by Urban PM Extracts—Model Studies

**Figure 3:**
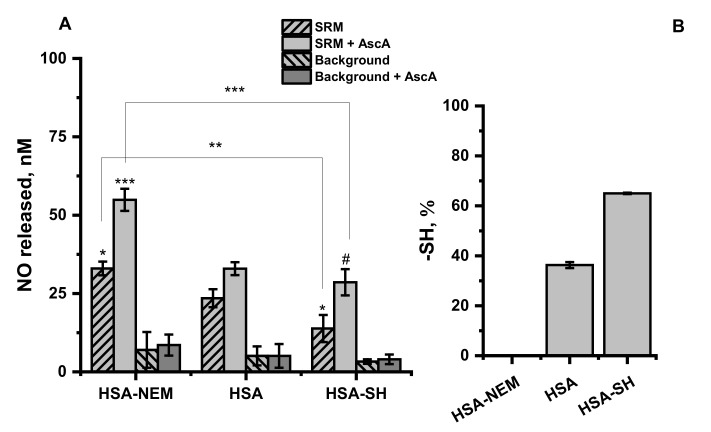
(**A**) The mean amount of NO released from GSNO in the presence of SRM aqueous extract =10% vol. with or without 1 µM of ascorbic acid (AscA) in the environment of 35 µM albumin with completely blocked thiol groups (HSA-NEM), unmodified albumin (HSA) and albumin with increased number of thiol groups (HSA-SH). Background refers to the lack of treatment with SRM extract. Measurements were performed in Tris buffer 0.1 M, pH = 7.4, T = 37 °C in the presence of 5 µM of GSNO, in the dark. Bars represent the means ± SD of three independent experiments. The values of * *p* < 0.05, ** *p* < 0.01 and *** *p* < 0.001 vs. controls, were considered significant; # *p* > 0.05. (**B**) The free thiol concentration determined in HSA-NEM, HSA and HSA-SH. Bars represent the means ± SD of three independent experiments.

Human serum albumin (HSA) possesses a free thiol group located at Cys34 and this protein is considered a major reservoir of free thiols in the blood plasma [56]. As was already shown the number of free thiol groups in plasma from CHFE and CAD patients is decreased (Figure 2) in comparison to healthy control. To verify the effect of the number of free thiol groups on the protective ability of albumin against GSNO decomposition induced by metal ions derived from the SRM extract, three types of HSA were used: HSA with completely blocked (HSA-NEM), unmodified (HSA), and increased thiol groups (HSA-SH) having 0, 36 and 65% of free -SH (Figure 3B). The amount of NO released from GSNO under the influence of aqueous SRM extract in the albumin environment is presented in Figure 3A.

Accessibility of a free thiol group in albumin had a significant impact on the amount of NO released from GNSO in the presence of SRM extract with and without the addition of AscA. HSA-NEM environment meaningfully enhanced GSNO decomposition in both cases (*p* = 0.01; *p* = 0.0007 for AscA and AscA free solution, respectively) in comparison to the unmodified albumin. Conversely, in the presence of HSA-SH, characterized by improved access to the free thiols, efficient reduction in GSNO decomposition upon SRM extract treatment (*p* = 0.03) was observed. When additionally, AscA was introduced, the observed decrease was nonsignificant compared to the unmodified HSA (*p* = 0.2) but significant compared to HSA-NEM (*p* < 0.001).

Albumin is considered a major extracellular antioxidant, common drug transporter and specific transition metal ions scavenger [57]. Cys34 group of albumin is significantly involved in the mentioned functions of this protein. The oxidized thiol group can be converted to sulfenic acid, then to disulfide with the potential to be redox cycled to its primary form (HSA-SH) in the reaction with other free thiols [57,58]. The thiol moiety is also highly reactive and capable to undergo nitrosylation (HSA-NO) resulting in the formation of S-nitrosoalbumin, which is more resistant against degradation by SRM extract than GSNO [37,40]. Furthermore, hydrogen peroxide or hydroxyl radical, potentially formed in the process of ascorbic acid oxidation catalyzed by copper ions delivered from SRM extracts, might be trapped by Cys34 of albumin [59]. The larger the pool of hydrogen peroxide or hydroxyl radical available, the higher the potential for exposure of GSNO to the uncontrolled release of NO [60]. Thus, the decreased number of free thiols on Cys34 simultaneously inhibits its ROS scavenging properties and slows down restoring of the reduced form [57,58], and might be one of the possible explanations for the observed decrease in NO release from GSNO with increased access to free -SH group on HSA.

### 2.5. Effect of Urban PM Extracts on Free Thiol Groups in Albumin and S-Transnitrosation Process—Model Studies

**Figure 4:**
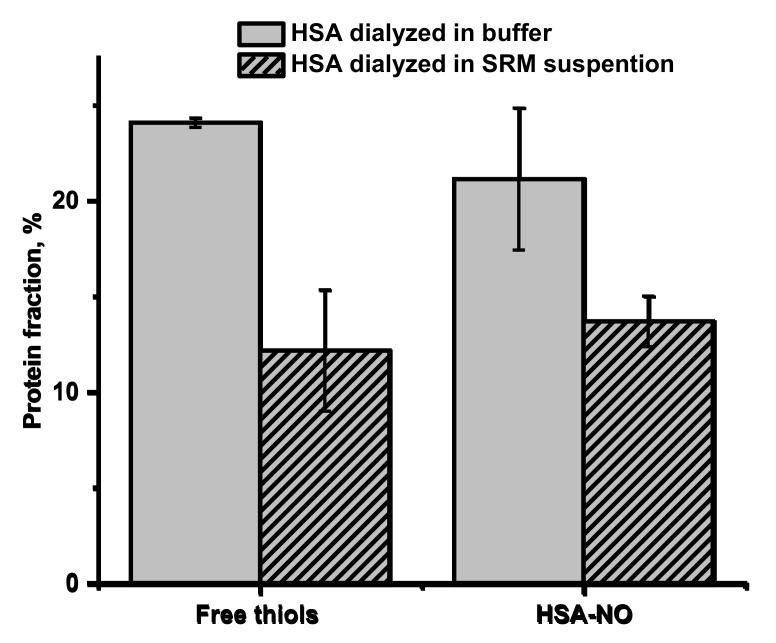
Free thiols groups in HSA and S-NO moieties in HSA (HSA-NO) resulting from S-trans-nitrosation of HSA using GSNO presented as percentage of protein fraction (plain gray: HSA dialyzed in Tris buffer pH 7.8, stripped gray: HSA dialyzed in buffered suspension of SRM). Bars represent the means ± SD of three independent experiments.

The decreased number of free thiols groups in albumin found in the plasma of CVD participants can be further modified by exposure to pollutants. It was shown that different adducts at the Cys34 locus of HSA are formed in patients with heart disease compared to healthy controls, and it is also influenced by exposure to urban air pollution [61]. There were also reports that HSA dialyzed in the presence of SRM, captured some metal ions from the bulk suspension (among others Al, Fe, Zn, Pb), and this process influenced albumin stability and negatively affected binding of warfarin and aspirin to it [39]. To check whether the extract from SRM can cause permanent changes in the availability of free thiol at Cys34 in HAS, 24-h dialysis of albumin with SRM suspension was performed. As shown in Figure 4, a considerable free thiol depletion in albumin was determined when HSA was dialyzed in the buffered suspension of SRM (from 24 to 12%), compared to dialysis in buffer alone. The process of dialysis itself resulted in a decrease in the free thiol concentration from 36% for a freshly prepared one (Figure 3) to 24% found after 24 h of dialysis in the buffer (pH 7.8), indicating that under the studied conditions, albumin’s Cys34 is susceptible to oxidation. Furthermore, apart from oxidation, Cys34 exhibits a high affinity to the metal ions, which can bind effectively via a single metal–sulfur bond [37].

The lower access to free thiol groups in HSA may influence the efficiency of the trans-S-nitrosation process and in consequence decrease S-nitrosoalbumin formation. It is postulated that S-nitrosoalbumin is mainly formed by the transfer of NO group from GSNO to reactive Cys34 thiol group in albumin [62]. As shown in Figure 4, the decrease in the number of free thiols in HSA corresponds directly to the decrease in the amount of formed HSA-NO. Since the trans-S-nitrosation process is very efficient, it leads to the nitrosation of all available free thiol groups. Thus, the free thiol groups on HSA act as determinants of HSA-NO formation, which makes depletion of free -SH groups caused by exposure to pollutants a factor that may lead to dysregulation of SNO homeostasis and contribute to CVD pathogenesis.

### 2.6. γ-Glutamyl Transpeptidase Activity in Human Blood Plasma and Its Influence on NO Release from GSNO

**Figure 5:**
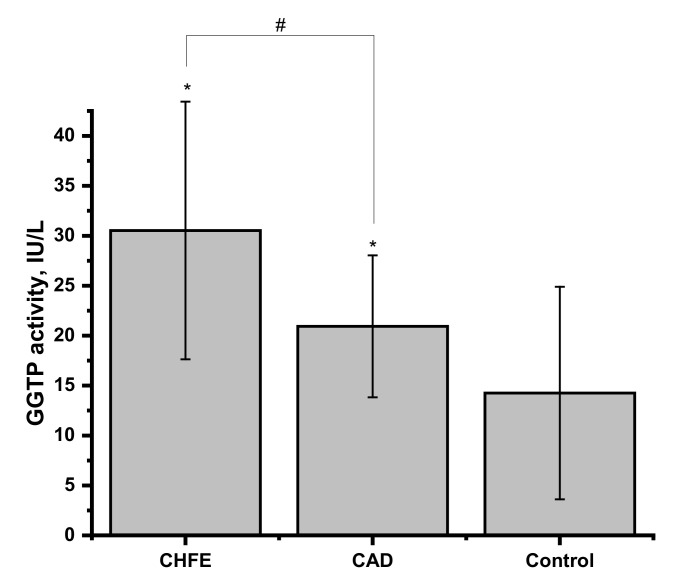
The assessment of γ-glutamyl transpeptidase (GGTP) activity in human plasma from CHFE (*n* = 7), CAD (*n* = 8) and control (*n* = 15) participants. All results are reported as the average of three-fold repeated measurements and the data represents mean ± SD. The values of * *p* < 0.05, were considered significant; # *p* > 0.05.

Cardiovascular diseases, in particular CHFE and CAD, are strongly associated with the elevated activity of the γ-glutamyl transpeptidase (GGTP) in blood plasma, the enzyme involved in the glutathione metabolism [25,26,27,28]. Hogg et al. reported that GGTP can use GSNO as a substrate to generate a less stable form, S-nitrosocysteinylglycine [63]. The activity of GTTP present in the studied human blood plasma samples was evaluated, and the results are presented in Figure 5. The group of CHFE patients has the highest activity value, but it is not significantly higher than in CAD (*p* = 0.2539). At the same time, results from healthy controls were significantly lower compared to both CVD groups (*p* = 0.0124 and *p* = 0.0291 for CHFE and CAD, respectively).

Furthermore, the strength and association between measured GGTP activity and the amount of NO released in human plasma of all participants and free thiols concentration were calculated using Pearson’s correlation test (Table 2). A medium to strong, positive, and significant relationship between GSNO decomposition and GGTP activity was found for all human plasma conditions. The addition of AscA to the SRM extract slightly reduced the Pearson’s correlation coefficient value while AscA supplementation of the neat plasma sample noticeably increased this value. In animal model studies, a correlation between the enzymatic activity of GGTP and the hydrolysis of S-nitrosoglutathione was demonstrated [64]. Furthermore, hydrolysis of GSNO was catalyzed by the presence of transition metal ions such as copper or iron [63,65]. The increased activity of GTTP in plasma from CVD patients may explain the registered increase in the NO released in this environment regardless of the addition of the SRM extract and/or AscA. The correlation between the concentration of the measured free thiol groups and GGTP activity was also verified. The calculation revealed a moderate, negative, and significant relationship between both parameters measured from human plasma samples. The primary biological function of GGTP is to break down extracellular GSH to generate γ-glutamyl compounds and cysteinyl-glycine to be used by cells for intracellular resynthesis of GSH. However, it is noteworthy that these intermediates are readily oxidizable compounds that can promote further oxidative damage, therefore they might act like limiting substrates in the synthesis of GSH and eventually lower the total -SH level in blood plasma. In the group of patients with metabolic syndrome, it was already shown that the elevated circulating levels of cysteinyl–glycine and cysteine derived from the hydrolysis of GSH are a consequence of the increased level of GGTP [66].

### 2.7. Metal Ions Content of Plasma Samples

Trace elements profiles for blood plasma samples collected from 28 study participants were determined to examine the differences in the ion balance between groups. Results are presented in Table 3. The plasma concentrations of the investigated elements are in good agreement with expected reference values. The decrease in the mean content of selenium among patients with CVD is supported by several meta-analyses, which have assessed the association between selenium and heart disease. In the human genome, there are identified 25 so-called selenoproteins with important functions in antioxidant defense, thyroid metabolism, protein folding, and immunity. One of them is selenoprotein P, the decrease of which has been correlated with greater risk of the first cardiovascular event, hospitalization, and mortality in the context of acute heart failure [67]. In addition, the significant drop in serum iron concentration in the CVD groups versus controls has been previously revealed, as the iron level is reduced by risk factors involved in the pathogenesis of CVD like inflammation and infection [68]. Similarly, elevated levels of heavy metals such as cadmium and lead in the blood serum in patients with CVD have been associated with atherosclerosis due to induced oxidative stress and inflammation as well as increased blood pressure by impairing nitric oxide signaling and reducing endothelium-dependent vasorelaxation [45]. The trace elements ICP-MS analysis of plasma samples for the studied groups allows assuming that the groups were completed in a consistent manner and that the observed difference in the content of several elements in CVD group in comparison to the control group is in line with the available literature on the subject. It should be mentioned that despite the lack of obvious trace element factors boosting the degradation of GSNO in plasma of the participants, chronic exposure to PMs may contribute to the exacerbation of the disturbances of metal ion homeostasis.

## 3. Materials and Methods

### 3.1. Materials

Unless indicated otherwise, all chemicals were purchased from Sigma Aldrich and were of the highest purity available. All solutions were prepared by using ultrapure water. SRM was supplied by the National Institute of Standards and Technology (NIST).

### 3.2. Patients and Sample Collection

For ex vivo analyses, fifteen patients with established significant coronary artery disease (CAD) or chronic heart failure exacerbations (CHFE) were enrolled to take part in this case-control study, after meeting the inclusion criteria and providing informed consent, according to the local internal review board rules. Study participants were recruited from the Department of Coronary Disease and Heart Failure, Jagiellonian University Medical College, John Paul II Hospital, Cracow, Poland. Both male and female patients were included consecutively if they were in the age range between 40–80 years old, nonsmokers, and hospitalized due to exacerbation of chronic heart failure or coronary artery disease who required intensification of treatment. A significant CAD was defined as coronary stenosis of 70% or greater in the epicardial coronary artery or of 50% or greater in the left main coronary artery, or when the fractional flow reserve was equal to or less than 0.80 in patients with borderline coronary lesions on angiography. Participants were excluded from the study due to positive history of myocardial infarction or stroke (within 1 month from the day of inclusion), diagnosis of type 2 or 1 diabetes, amyloidosis, Crohn’s disease, or nephrotic syndrome. The group of healthy volunteers was recruited from the Faculty of Chemistry of Jagiellonian University after meeting the same inclusion criteria with an indication of the lack of diagnosed cardiovascular diseases.

Demographic characteristics of controls and patients are described in Table 4, while the biochemical parameters and pharmacological treatment for patients are given in Table 5. For all participants, 9 mL of venous blood samples were collected into Vacuette sodium citrate 3.2% containing tubes. Next, they were centrifuged for 10 min at 200× *g* using a refrigerated centrifuge. The resulting supernatant was stored at −20 °C until assay.

### 3.3. Ethics

The study was approved by the Jagiellonian University Bioethics Committee (approval number: 1072.6120.214.2019 to M.B.). Studies were conducted in accordance with the Declaration of Helsinki. Each study participant provided written informed consent before enrollment.

### 3.4. GSNO Preparation

GSNO was synthesized based on the method described by Hart [67]. Equimolar solutions of GSH and sodium nitrite were mixed in the presence of hydrochloric acid and stirred vigorously for 5 min in the dark. After that time, the pH of the red solution was adjusted to neutral by the addition of concentrated NaOH.

### 3.5. Particulate Matter’s Extract Preparation

SRM, a standard particulate matter (PM), at a ratio of 5 mg/mL was suspended in 0.1 M Tris buffer, pH = 7.8 and shaken for 24 h at room temperature. After this time, suspensions were filtered through molecular weight cut-off membrane-based centrifugal filters (Amicon Ultra-4, MWCO 100 kDa). The metal content of the SRM extract solution was recently reported by us [40] and was also given in the Supplementary Materials.

### 3.6. Human Serum Albumin Modification

Albumin from human serum, HSA (fatty acid free, globulin free, ≥99%) was used without further purification. For model studies it was presumed that the average concentration of albumin in human plasma was 700 µM. Modification of thiol group were obtained by incubation of 0.7 mM HSA in Tris buffer 0.1 M, pH = 7.8 with 7 mM N-ethylmaleimide (HSA-NEM) or glutathione (HSA-SH) for 30 min at 37 °C. After this time, remaining reagents were removed using a molecular weight cut-off membrane-based centrifugal filter (Amicon Ultra-4, MWCO 30 kDa). Exposure of human serum albumin to particulate matter was achieved by using the dialysis technique. In a typical experiment HSA dissolved in Tris buffer 0.1 M, pH = 7.8, at 10 mg/mL concentration was placed in the Pur-A-Lyzer Dialysis Tube, 3.5 kDa MWCO (Sigma-Aldrich). The dialysis was carried out for 12 h in 40 mL of SRM suspension at 2 mg/mL concentration. It was followed by dialysis in a fresh portion of Tris buffer for another 12 h. Treated protein was collected and stored frozen.

### 3.7. Determination of NO Release from GSNO

NO release was monitored with an amino-700 nitric oxide sensor connected to an inNO-T nitric oxide measuring system (Innovative Instruments, Tampa FL). The electrode was immersed in a double-wall heated beaker connected with a thermostat set to 37 °C. In a typical experiment, 5 mL of solution containing the blood plasma diluted 20-fold with buffer without any additions or with 10% of SRM extracts were prepared and strictly protected from light. After the stabilization of the electrode response, an aliquot of exogenous GSNO (5 µM) was added. To study the effect of ascorbic acid (AscA), an aliquot of AscA (1 µM) was added just before the injection of GSNO. The electrode was calibrated at 37 °C, by measuring the signal increase after adding the standard solutions of NaNO_2_ to 0.6 mM KI with 0.1 M H_2_SO_4_.

### 3.8. Determination of Free -SH Groups

Content of free -SH groups in human plasma, in freshly prepared HSA or protein modified by dialysis or the reduction (HSA-NEM, HSA-SH) was measured with Ellman’s reagent, 5,5′-dithiobis(2-nitrobenzoic acid) (DTNB), using a molar extinction coefficient of 13,600 M^−1^cm^−1^ at 412 nm. DTNB was kept at a 10-fold molar excess over the protein concentration.

### 3.9. Transnitrosation of HSA

HSA (0.4 mM) was incubated with 4 mM GSNO for 15 min. After that time, unreacted thiol groups were blocked with 4 mM N-ethylmaleimide. The remaining low-molecular-weight reagents were removed with molecular cut-off filters and the aliquots of HSA-NO solutions were stored in the freezer at −70 °C.

### 3.10. Determination of HSA-NO Concentration

The amount of S-NO moieties per HSA molecule was determined using a modified Saville assay [40,68]. HSA-NO was added to the solution containing 0.04 M HCl, 0.1% HgCl_2_, 0.1% sulfanilamide and 0.01% N-(1-naphtyl)ethylenediamine and incubated at room temperature for 15 min. The addition of aqueous mercury (Hg^2+^) results in NO release, which in acidic conditions undergoes a reaction with O_2_ to form NO_2_^−^. Nitrite through a reaction with sulphanilamide forms a diazonium cation intermediate that couples with N-(1-naphthyl)ethylenediamine to form an azo dye (λ_max_ = 540 nm), which was followed at 540 nm. Amount of NO moieties in HSA-NO was estimated based on the calibration curve prepared with NaNO_2_.

### 3.11. γ-Glutamyltranspeptidase (GGTP) Activity

Plasma γ-GGTP activity level was measured by colorimetric assay kit (MAK089; Sigma-Aldrich) at 37 °C according to the manufacturer’s protocol. Briefly, the activity was determined by a coupled enzyme assay, in which the GGTP transfers the γ-glutamyl group from the substrate L-γ-Glutamyl-p-nitroanilide, liberating the chromophore *p*-nitroanilide. Absorbance measured at 418 nm was proportional to the GGTP activity.

### 3.12. ICP-MS Analysis of Plasma Samples

For sample preparation, 50 μL of human blood plasma were mixed with 100 μL of 65% HNO_3_ and 50μL of H_2_O_2_. The mixture was heated and shaken for 2 h at 65 °C until all denatured proteins were digested, and a clear solution was obtained. The digested solution was diluted with 4.15 mL of deionized water to give a final nitric acid concentration of 1.5%. This solution was used directly for the ICP-MS measurement. Calibration was carried out by external calibration using concentration ranges according to the expected elemental plasma values (Table 6). Multielement standard solution 5 for ICP (Merck, no. 54704, certified reference material *Trace***CERT**) was diluted with 1% HNO_3_ and for each element at least six calibration points were considered for calculation. The method validation test was performed with a certified standard reference material, namely, ClinChek^®^ -Control Serum Control lyophilized level I. The serum samples were prepared as previously described and the results with control range are presented in Table 7. Only those elements for which the confidence intervals were in line with or close to the reference concentration range were included.

### 3.13. Statistics

All data were analyzed by using Statistica 13 PL software, and are presented as means ± SD of individual samples. For measurements of NO release from GSNO in the blood plasma environment based on our model study, we anticipated that minimum three participants per group were required to determine the mean differences across two groups (α = 0.05, [Power = 90%], two-sided. Statistical comparisons between groups were performed by student *t*-test. Examination of the strength of an association between two parameters were conducted using Pearson’s correlation test. Probabilities of *p* < 0.05 were considered as statistically significant. The following notification is used * *p* < 0.05, ** *p* < 0.01, *** *p* < 0.001.

## 4. Conclusions

**Figure 6:**
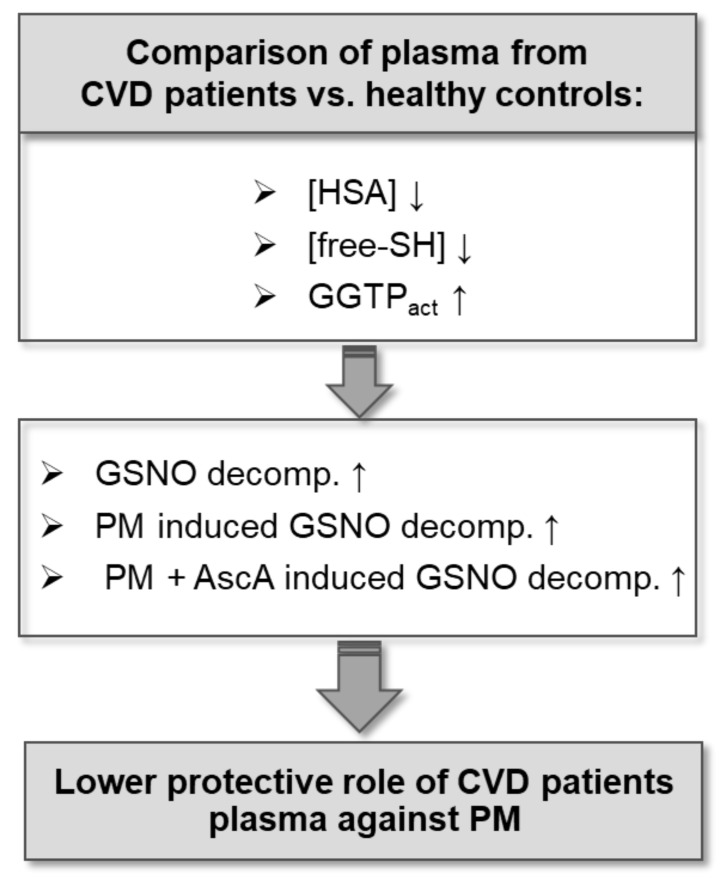
Schematic representation of the major outcomes. Plasma from cardiovascular diseases (CVD) patients compared to healthy control is characterized by the decreased concentration of human serum albumin (HSA), lowered availability of free thiol groups (SH), and increased activity of gamma-glutamyltransferase (GGTP). These differences may be responsible for increased decomposition of S-nitrosoglutathione (GSNO), particularly under the influence of particulate matter (PM) and the supplementation with ascorbic acid (AscA).

Numerous epidemiological studies emphasize that exposure to air pollution increases the risk of cardiovascular events. At the same time, the deciphering of the precise mechanisms involved in CVD pathophysiology is still needful. The results of the preliminary case-control studies presented herein demonstrate a novel approach in the investigation of the interplay of aqueous PM extract and CVD progression and are briefly summarized in Figure 6. The investigation of S-nitrosoglutathione decomposition by PM-derived metal ions has shown that in the blood plasma environment of patients with ongoing exacerbation of heart failure or coronary artery disease, the level of the released NO was higher in comparison with a group of healthy volunteers. This process was faster and more pronounced after ascorbic acid supplementation for the CVD groups indicating a lower protective ability of their plasma against the metal ions activity. Multiple regression analysis of γ-glutamyl transpeptidase activity and free thiol groups concentration points to them as differentiating factors in protective plasma activity between the studied patients and healthy volunteers. The relevance of Cys34 accessibility in albumin was supported by the model studies influencing both PM-inducing GSNO decomposition and the S-transnitrosation process. These studies broaden our understanding of the impact of urban PM on the progression of cardiovascular diseases by disrupting biological signaling in the S-nitrosation pathways. Our study, apart from showing the negative influence of the inorganic fraction of PM on the progression of cardiovascular diseases, indicated the impaired protective ability of blood plasma taken from people with CVD against PM. Correlation between the changes in plasma constituents induced by such diseases and the long-term exposure to PMs on the NO-carriers formation and decomposition equilibria need to be studied more extensively, including a larger group of people. Another challenge would be to find more checkpoints enabling assessment of such deteriorating effects on NO signaling pathways. The results of this research uphold the idea to pay attention to the inorganic fraction of urban PM as one of the environmental factors causing or leading to the progression of cardiovascular disease. Further research should be focused on searching the clinical indicators that would associate the harmful effects of PM with the development of pathophysiological states that may result in cardiovascular diseases.

## Figures and Tables

**Table 1:** Pearson’s correlation coefficient between free thiol groups concentration and GSNO decomposition measured in neat blood plasma samples (blank) or supplemented with particulate matter’s extract (SRM) or/and ascorbic acid (AscA). The values of *p* < 0.05 were considered significant.

	R	*p*
SRM	−0.3871	0.035
SRM + AscA	−0.7477	<0.001
Blank	−0.3005	0.107
Blank + AscA	−0.7070	<0.001

**Table 2:** Pearson’s correlation coefficient between γ-glutamyl transpeptidase (GGTP) activity and GSNO decomposition measured in neat blood plasma samples (blank) or supplemented with particulate matter’s extract (SRM) or/and ascorbic acid (AscA) as well as free thiol groups (free -SH). The values of *p* < 0.05 were considered significant.

	R	*p*
SRM	0.66961	<0.001
SRM + AscA	0.40602	0.029
Blank	0.61778	<0.001
Blank + AscA	0.72515	<0.001
Free -SH	−0.45181	0.014

**Table 3:** ICP-MS trace element analysis: results of 28 human plasma trace element analyses.

Element	CHFE Patients (*n* = 7)	CAD Patients (*n* = 8)	Controls (*n* = 13)	Range
	Mean ± Std Dev.	Mean ± Std Dev.	Mean ± Std Dev.	
Be (μg/L)	1.09 ± 0.43	0.87 ± 0.24	0.94 ± 0.08	0.69–1.38
Mn (μg/L)	3.82 ± 2.16	4.32 ± 3.17	3.73 ± 2.25	1.46–7.75
Co (μg/L)	1.22 ± 0.61	1.16 ± 0.62	1.23 ± 0.56	0.59–1.85
Se (μg/L)	58.52 ± 3.77 **	69.13 ± 1.05	74.35 ± 1.62	55.40–75.88
Ag (μg/L)	1.41 ± 0.39	1.3 ± 0.27	1.12 ± 0.37	0.98–1.74
Cd (μg/L)	0.84 ± 0.19 **	0.54 ± 0.08	0.52 ± 0.08	0.43–1.04
Cs (μg/L)	0.88 ± 0.03	0.58 ± 0.03	0.68 ± 0.02	0.55–0.91
Ba (μg/L)	8.01 ± 3.14	5.56 ± 1.59	10.22 ± 5.82	4.44–14.33
Tl (μg/L)	0.07 ± 0.04	0.04 ± 0.01	0.03 ± 0.01	0.02–0.11
Pb (μg/L)	9.98 ± 0.04 *	9.70 ± 0.49 ***	7.97 ± 0.12	7.88–11.24
Bi (μg/L)	1.94 ± 0.87	1.68 ± 1.33	2.18 ± 1.45	0.98–3.85
Ni (μg/L)	4.83 ± 3.57	4.13 ± 2.64	4.64 ± 3.34	0.78–8.15
Fe (mg/L)	1.16 ± 0.15*	1.12 ± 0.03 **	1.78 ± 0.20	1.01–2.00
Cu (mg/L)	1.16 ± 0.16	1.01 ± 0.19	1.02 ± 0.19	0.80–1.21
Zn (mg/L)	0.65 ± 0.12	0.73 ± 0.08	0.70 ± 0.12	0.51–0.822

The values of * *p* < 0.05, ** *p* < 0.01 and *** *p* < 0.001 vs. controls, were considered significant.

**Table 4:** Demographic data of participants: age range, the mean age, males/females ratio and body mass index (BMI) reported for healthy controls and in patients with chronic heart failure exacerbations (CHFE) or coronary artery disease (CAD).

Subjects	*n* = 30	Age Range (years)	Mean Age (mean ± SD)	Males/Females
Healthy Controls	15	44–59	49 ± 5	11/4
Patients				
CHFE	7	53–76	68 ± 9	5/2
CAD	8	61–79	73 ± 6	7/1

**Table 5:** Laboratory and pharmaceutical data of patients with chronic heart failure exacerbations (CHFE) or coronary artery disease (CAD).

	CHFE	CAD	*p* Value
**Laboratory Investigaitons**	**Mean ± SD**		
HGB (g/dL)	13.20 ± 1.66	13.40 ± 1.42	>0.05
CREA (µmol/L)	99.86 ± 20.67	91.75 ± 10.95	>0.05
eGFR (mL/min/1.73 m^2^)	65.86 ± 23.38	69.75 ± 7.43	>0.05
LDL-C (mmol/L)	2.68 ± 0.98	2.68 ± 0.82	>0.05
HDL-C (mmol/L)	1.06 ± 0.25	1.19 ± 0.22	>0.05
Non-HDL (mmol/L)	2.77 ± 0.95	2.90 ± 0.81	>0.05
TC (mmol/L)	3.83 ± 1.13	4.13 ± 0.67	>0.05
TG (mmol/L)	1.02 ± 0.37	1.68 ± 0.76	>0.05
EF (%)	21 ± 0.05	60 ± 0.05	<0.001
AIP	−0.03 ± 0.18	0.11 ± 0.25	=0.02
CRI-I	3.59 ± 0.61	3.63 ± 1.05	>0.05
**Pharmacological Treatment**	**CHFE**	**CAD**	
Statins (%)	57.14	87.50	
ACEIs or ARB (%)	57.14	100	
β -Blockers (%)	100	87.50	
CCBs (%)	0	37.50	
Diuretics (%)	100	75.00	
Antiplatelet agents (%)	100	100	

Abbreviations: HGB hemoglobin, CREA creatinine, eGFR estimated glomerular filtration rate, LDL-C low-density lipoprotein cholesterol, HDL-C high-density lipoprotein cholesterol, TC total cholesterol, TG triglyceride, AIP atherogenic index of plasma, Castelli risk index-I, ACEIs angiotensin -converting enzyme inhibitor, ARB angiotensin-receptor blocker, CCB channel calcium blocker

**Table 6:** Calibration range and ICP-MS modes for analyzed elements performed on NexION 2000C (Perkin Elmer).

Element	Calibration Range [μg/L]	ICP-MS Mode
Be	0.01–10	He
Mn	0.01–10	He
Co	0.01–10	He
Se	0.01–10	CH_4_
Ag	0.01–10	No Gas
Cd	0.01–10	No Gas
Cs	0.01–10	No Gas
Ba	0.01–10	No Gas
Tl	0.01–10	No Gas
Pb	0.01–10	He
Bi	0.01–10	He
Fe	0.5–100	He
Ni	0.01–10	He
Cu	0.01–10	He
Zn	0.01–10	He

**Table 7:** Analysis of reference materials, ClinChek®—control trace elements.

Element	Unit	Range of Measured Concentrations	Reference Range of Concentrations
Be	µg/L	1.52–1.93	1.61–2.42
Mn	µg/L	1.16–1.66	1.81–2.72
Co	µg/L	2.25–2.75	1.74–2.61
Se	µg/L	96.44–129.4	98.1–147
Ag	µg/L	4.43–4.84	3.98–5.97
Cd	µg/L	1.26–1.85	1.50–2.25
Ba	µg/L	390.25–457.61	364–492
Tl	µg/L	1.74–1.90	1.49–2.23
Bi	µg/L	0.77–1.10	0.79–1.20
Fe	mg/L	1.15–1.49	0.874–1.31
Ni	µg/L	1.08–2.24	1.54–2.86
Cu	mg/L	0.83–1.3	0.902–1.22
Zn	mg/L	0.53–0.85	0.626–0.85

## Data Availability

The data presented in this study are available in the main text.

## Supplementary Materials

The following are available online at https://www.mdpi.com/article/10.3390/ijms221910500/s1.
