# Supplementary material for: Blood Plasma’s Protective Ability against the Degradation of S-Nitrosoglutathione under the Influence of Air-Pollution-Derived Metal Ions in Patients with Exacerbation of Heart Failure and Coronary Artery Disease
Source: Int J Mol Sci. 2021 Sep 28;22(19):10500. doi: 10.3390/ijms221910500 (PMC8508800; doi:10.3390/ijms221910500)
Supplement: Supplementary file 1 [file ijms-22-10500-s001.zip › ijms-1382558-supplementary.pdf]

Blood plasma protective ability against the degradation of S-nitrosoglutathione under the influence of air pollution-derived metal ions in patients with exacerbation of heart failure and coronary artery disease

Anna Wądołek [1], Dominika Drwiła [2], Maria Oszajca [1], Grażyna Stochel [1], Ewa Konduracka [3,*] and Małgorzata Brindell [1,*]

[1] *Faculty of Chemistry, Jagiellonian University in Kraków, Gronostajowa 2, 30-387 Krakow, Poland anna.wadolek@doctoral.uj.edu.pl (A.W.), stochel@chemia.uj.edu.pl (G.S.)*
[2] *Department of Coronary Disease and Heart Failure, John Paul II Hospital, Krakow, Poland dominika.drwila@gmail.com (D.D)*
[3] *Jagiellonian University Medical College, Department of Coronary Disease and Heart Failure, John Paul II Hospital, Krakow, Poland*
* Correspondence: maria.oszajca@uj.edu.pl (M.O.); ewa.konduracka@uj.edu.pl (E.K.); malgorzata.brindell@uj.edu.pl (M.B)

Table S1. ICP-OES analysis of the SRM 1648a extract solution in Tris buffer 0.1 M, pH = 7.4 (data taken from ChemSusChem 2019, 12, 661 – 671, http://doi.org/10.1002/cssc.v12.3.)

Element	$mg \cdot L^{-1}$
Fe	4.10
Sb	2.56
Zn	2.46
Cu	0.98
Ti	0.92
Mn	0.69
Mo	0.44
Sn	0.44
Hg	0.35
Ni	0.20
Cd	0.07
Cr	0.06
